# Sensitivity and Specificity of the World Health Organization Dengue Classification Schemes for Severe Dengue Assessment in Children in Rio de Janeiro

**DOI:** 10.1371/journal.pone.0096314

**Published:** 2014-04-28

**Authors:** Gleicy A. Macedo, Michelle Luiza C. Gonin, Sheila M. Pone, Oswaldo G. Cruz, Flávio F. Nobre, Patrícia Brasil

**Affiliations:** 1 Laboratório de Engenharia em Sistemas de Saúde, Programa de Engenharia Biomédica/COPPE/UFRJ, Rio de Janeiro, Brasil; 2 Hospital Municipal Jesus (HMMJ) –SMSDC-RJ, Rio de Janeiro, Brasil; 3 Fundação Oswaldo Cruz, Rio de Janeiro, Brasil; Duke-National University of Singapore Graduate Medical School, Singapore

## Abstract

**Background:**

The clinical definition of severe dengue fever remains a challenge for researchers in hyperendemic areas like Brazil. The ability of the traditional (1997) as well as the revised (2009) World Health Organization (WHO) dengue case classification schemes to detect severe dengue cases was evaluated in 267 children admitted to hospital with laboratory-confirmed dengue.

**Principal Findings:**

Using the traditional scheme, 28.5% of patients could not be assigned to any category, while the revised scheme categorized all patients. Intensive therapeutic interventions were used as the reference standard to evaluate the ability of both the traditional and revised schemes to detect severe dengue cases. Analyses of the classified cases (n = 183) demonstrated that the revised scheme had better sensitivity (86.8%, *P*<0.001), while the traditional scheme had better specificity (93.4%, *P*<0.001) for the detection of severe forms of dengue.

**Conclusions/Significance:**

This improved sensitivity of the revised scheme allows for better case capture and increased ICU admission, which may aid pediatricians in avoiding deaths due to severe dengue among children, but, in turn, it may also result in the misclassification of the patients' condition as severe, reflected in the observed lower positive predictive value (61.6%, *P*<0.001) when compared with the traditional scheme (82.6%, *P*<0.001). The inclusion of unusual dengue manifestations in the revised scheme has not shifted the emphasis from the most important aspects of dengue disease and the major factors contributing to fatality in this study: shock with consequent organ dysfunction.

## Introduction

Dengue is the most widely distributed viral hemorrhagic fever in the tropical world, annually infecting approximately 100 million people in Southeast Asia, the Pacific region, and the Americas and often causing epidemics in urban and peri-urban areas [1]. In 2013, 2,351,703 cases were reported in America. Brazil was responsible for approximately 61% of these cases (1,451,432 cases), and all 4 serotypes of the dengue virus have been isolated in almost all Brazilian states [2].

Severe forms of dengue disease were first recognized in the 1950 s during dengue epidemics in the Philippines and Thailand. Today, severe dengue affects most Asian and Latin American countries and has become a leading cause of hospitalization and death among children in these regions [3]. An estimated 500,000 people with severe dengue require hospitalization each year, a large proportion of whom are children; approximately 2.5% of those affected die. In Brazil, the increase in hospitalizations and deaths among children has become a problem in recent years [4].

Although dengue is a single disease entity, it has various clinical presentations and often has an unpredictable clinical pathogenesis and outcome [5]. Patients with dengue can present with a range of clinical symptoms that varies according to its severity (asymptomatic, mild, or severe) and the age group affected (children or adults). To describe and categorize the common manifestations of dengue, the World Health Organization (WHO) developed a classification system that evolved from pioneering studies in Thailand in the 1950 s and 1960 s. This guideline for control, diagnosis, clinical classification, and treatment of dengue was first proposed in 1975 and revised in 1997, on the basis of a clinical study of 123 Thai children in 1966 [6], [7]. It grouped the clinical presentations of dengue disease as dengue fever (DF), dengue hemorrhagic fever (DHF), and dengue shock syndrome (DSS). DHF is divided into 4 grades. Grades I and II are classified as DHF, and grades III and IV are considered DSS.

Nevertheless, some studies have shown that applying this classification system is challenging in dengue-endemic areas. The appearance of different manifestations such as dengue with hemorrhage but without plasma leakage or dengue with shock but without fulfilling the 4 DHF criteria (fever lasting 2–7 days, a tendency for hemorrhage shown by a positive tourniquet test or spontaneous bleeding, thrombocytopenia ≤100,000 platelets/mm^3^, and evidence of plasma leakage), poses difficulties to clinicians in applying the current case classification scheme. The major problems identified were the rigidity of the definitions, low sensitivity, and difficulty experienced by some clinicians to differentiate DHF from DF since the clinical and basic laboratory parameters overlap in some cases [8]–[11]. To address these difficulties, the WHO Dengue Scientific Working Group has designed a multicenter study, DENCO (Dengue Control), to evaluate the perceived limitations of the WHO 1997 dengue classification scheme in all age groups from Southeast Asia and Latin America [12]. Based on the findings of this working group, a new classification scheme was proposed in 2009, which divides cases into dengue without warning signs, dengue with warning signs, and severe dengue [5].

The recent dengue epidemics among children in Rio de Janeiro provide an opportunity to assess the ability of these WHO dengue classifications to identify effectively severe dengue cases. The aim of this study was to evaluate the sensitivity and specificity of the WHO 1997 dengue classification compared with the WHO 2009 dengue classification to assess severe dengue among children who were admitted to pediatric reference hospitals in Rio de Janeiro during the epidemics of 2007/2008 and 2010/2011, using the need for intensive care as a reference standard of severity.

## Methods

### Data set and data management

A hospital-based study was performed in 3 tertiary care centers for children during the dengue epidemics of November 2007 through May 2008 and November 2010 through May 2011, in Rio de Janeiro, Brazil. These hospitals were part of the dengue network study whose regional reference center was the Laboratório de Doenças Febris Agudas at Instituto Evandro Chagas (IPEC/FIOCRUZ).

The sources of data were the computerized medical records collected from databases from 3 centers, all of which utilized a standardized protocol with demographic, clinical, and laboratory assessments, including daily hematocrit and platelet counts, serological findings, and therapeutic information. All cases were retrospectively reviewed by specialists to ensure data consistency and classify the cases according to the traditional (1997) and current (2009) WHO schemes [5], [7].

### Eligibility criteria

Inclusion criteria were children between 0 and 18 years of age who were admitted during the dengue epidemics of 2007/2008 and 2010/2011 to 1 of the 3 pediatric reference hospitals in Rio de Janeiro. Exclusion criteria were children without complete protocol data and laboratory-dengue confirmation.

### Case classification

#### WHO 1997 scheme

According to traditional scheme the cases were classified as dengue fever (DF), dengue hemorrhagic fever (DHF) and dengue shock syndrome (DSS).

DF was defined as laboratory-confirmed cases with high fever without evidence of plasma leakage, with or without hemorrhagic manifestation. DHF grades I and II were characterized by evidence of plasma leakage associated with the presence of hemorrhagic manifestations (petechiae, ecchymosis, purpura, or bleeding from the mucosa of the gastrointestinal or urinary tract, injection sites, or other locations) and thrombocytopenia (≤100,000 platelets/mm^3^) without shock. DSS was characterized by signs of circulatory failure, cold clammy skin, cyanosis, rapid pulse, pulse pressure <20 mmHg, or hypotension in the presence of a hemorrhagic manifestation [7].

Children with laboratory-confirmed dengue who had evidence of plasma leakage but did not comply with the criteria for DHF or DSS were defined as unclassified cases.

#### WHO 2009 scheme

According to new scheme proposed, cases were grouped into dengue without warning signs, dengue with warning signs, and severe dengue [5].

Warning signs included: abdominal pain or tendeness (not intermittent); persistent vomiting (more than 5 times in 6 hours or more than 3 times in 1 hour); clinical fluid accumulation including pleural effusion and ascites identified as a reduction of vesicular murmur or reduction of thoracic-vocal trill; abdominal distention or dullness decubitus, confirmed by abnormal imaging findings (chest radiography, thoracic and abdominal ultrasound, or computed tomography for pleural effusion and ascites or gallbladder wall thickening); mucosal hemorrhage (gastrointestinal hemorrhage and/or metrorrhagia); lethargy (alteration of consciousness and/or Glasgow score <15) or irritability; and liver enlargement (>2 cm below the costal margin). Laboratory findings were defined as follows: thrombocytopenia (platelet count <50,000/mm^3^) and hematocrit change of 20%, either raised or decreased by 20% from the baseline value during the convalescent period.

Severe dengue was defined by the following characteristics:

(i) Plasma leakage resulting in shock or fluid accumulation with respiratory distress (defined as respiratory discomfort, dyspnea, respiratory failure, or increased respiratory rate of >60 breaths/min for ages <2 months; >50 breaths/min for ages 2 months to 1 year; >40 breaths/min for ages 1 to 5 years; >30 breaths/min for ages 5 to 8 years; and >20 breaths/min for those older than 8 years). Shock was defined as the presence of at least 2 of the clinical signs of hypoperfusion (e.g., slow capillary filling, cold clammy skin, and rapid and weak pulse), with or without an associated weak pulse pressure (≤20 mm Hg) or hypotension for the specified age (decrease in blood arterial systolic pressure <5th percentile for age [<PAS5], calculated as age [years] ×2+70) [13]; or
(ii) severe bleeding (in this study, defined as patients who presented persistent and or severe overt bleeding in the presence of unstable hemodynamic status, regardless of the hematocrit level or required transfusion of blood products); or
(iii) severe organ involvement, e.g., severe hepatitis (aspartate aminotransferase/alanine aminotransferase levels ≥1000 IU/L), encephalitis (central nervous system involvement with impaired consciousness), or myocarditis (heart dysfunction, characterized by cardiac failure confirmed by echocardiography) and renal impairment (serum creatinine levels ≥2 times the upper limit of normal or a 2-fold increase from the baseline creatinine levels);

Multiple-organ dysfunction syndrome was considered when dysfunction involved ≥2 organs [14].

Cases were considered severe when classified as DSS by the traditional scheme and as severe dengue by the revised classification.

### Laboratory confirmation

Children who were admitted to 1 of the 3 hospitals during a dengue epidemic had at least 1 specific laboratory test performed. Cases were considered laboratory-confirmed dengue if dengue virus RNA was detected by reverse transcriptase polymerase chain reaction, IgM anti-dengue antibodies were detected from the third day after the onset of fever, or the non-structural protein-1 antigen test was positive. The dataset consisted of patients with laboratory-confirmed dengue. Others laboratories data included a minimum of 2 complete blood count analyses (hematocrit and platelet count) from separate days, blood chemistry values, and imaging (radiography, ultrasonography, computed tomography scan, and echocardiography). Complete blood count analyses were performed daily and imaging studies were carried out according to clinical demand to investigate the presence of fluid accumulation or clinical improvement.

### Reference standard

Deaths and intensive care unit (ICU) intervention were used as the reference standard to identify severe cases and, consequently, as the reference standard to compare both WHO classifications. Patients who required colloid, vasoactive amines, inotropic drugs, or transfusion of blood products; who utilized any kind of dialysis, or who required either invasive or non-invasive ventilator support were classified as having received ICU intervention.

### Statistical analysis

The traditional and revised schemes were compared according to their positive and negative predictive values, sensitivity, and specificity. Sensitivity is the probability that the diagnostic instrument, here the WHO classifications schemes, indicates a positive result for individuals with severe disease, and specificity is the probability of a negative result of the instrument for those who do not have severe disease. Individuals in whom the WHO classification was contrary to the class they belonged to were defined as false-negative or false-positive [15]. The positive predictive value for the test population is the probability that a person has the severe form of disease given that the test is positive. The negative predictive value for the test population is the probability that a person does not have severe disease when the test is negative.

To compare the differences between the sensitivity and specificity of the 2 classification schemes, a binomial test was applied, and 95% confidence intervals were obtained [16]. To analyze the positive and negative predictive values, the relative values were calculated and compared according to the method of Moskowitz and Pepe [17]. Patients who could not be classified according to either the traditional or the revised scheme were not included in the analyses. All statistical analyses were performed using the statistical software R 3.0.1 [18].

### Ethics statement

The Research Ethical Committee of IPEC/Fundação Oswaldo Cruz (Protocolo de pesquisa: 61/08. CAAE: 37230000009-08) and Secretaria Municipal de Saúde e Defesa Civil do Rio de Janeiro, RJ (SESDEC-RJ) (Protocolo de pesquisa: 33/09 CAAE: 00290314011-09) reviewed and approved this study. Informed consent was not obtained, as patient records/information was anonymized and de-identified prior to analysis.

## Results

**Figure 1:**
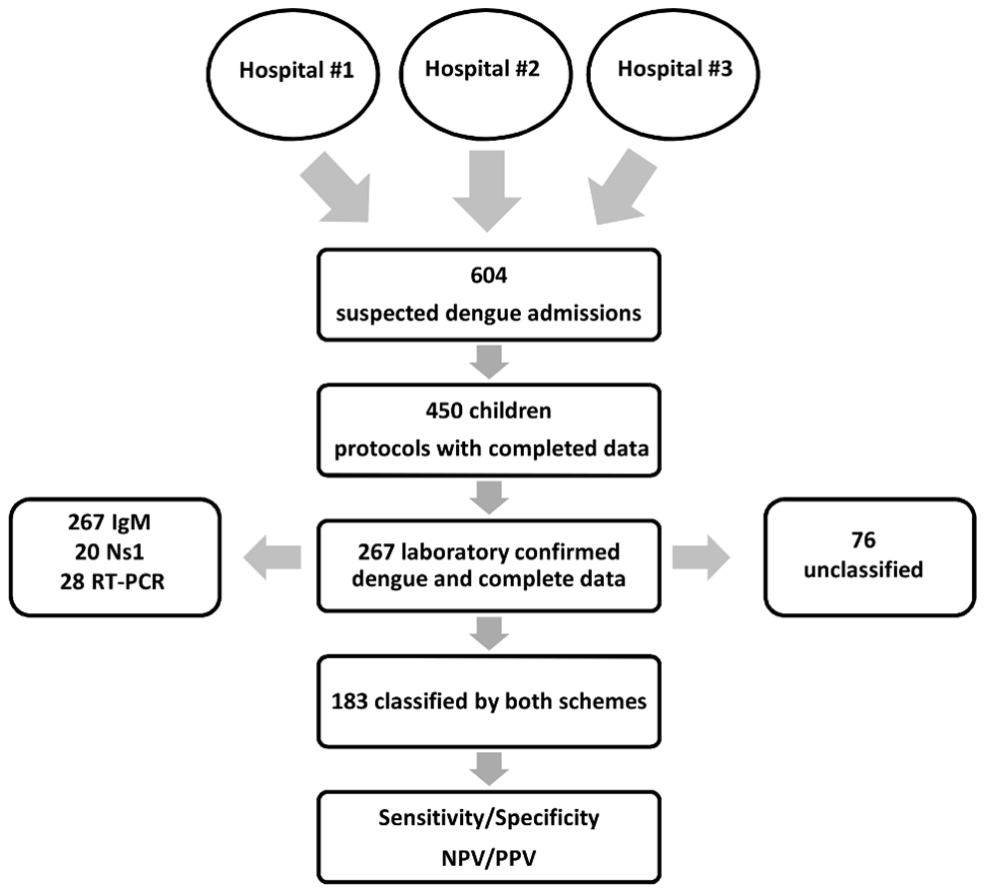
Flow diagram.

During the epidemics of 2007/2008 and 2010/2011, of 604 admissions to the reference hospitals, 450 children had complete set of clinical and laboratory data and 267 (59.3%) had laboratory-confirmed dengue. Of these 267 cases, 28 were RT-PCR positive, 267 were IgM positive, and 20 NS1 positive cases as shown in Figure 1. According to the revised scheme, 18 (6.7%) of the children with laboratory-confirmed dengue were classified as having dengue without warning signs, 142 (53.2%) as having dengue with warning signs, and 107 (40.1%) as having severe dengue. According to the traditional scheme, 26 (9.7%) of the 267 children were classified as DF, 119 (44.6%) as DHF, 46 (17.2%) as DSS, and 76 (28.5%) could not be classified (Table 1). The ages of children ranged from 0 to 18 years, with a median age of 8 years (interquartile range: 6–11), with a slightly higher proportion of girls (52.4%, 140/267) than boys (47.6%, 127/267). Eight cases were fatal (3%), all of which developed to severe dengue according to the revised scheme. The traditional scheme classified 6 of the 8 fatal cases (75%) as DSS and 1 case (12.5%) as DHF due to hemorrhagic complications. One fatal case with shock without hemorrhagic manifestations could not be classified into any specific category (Table 1). The median duration of hospitalization was 4 days (interquartile range: 2–6 days), with a maximum of 21 days. All dengue-related deaths occurred within the first 6 days of disease (Table 1). In terms of the days after fever onset, the patients were hospitalized on the fifth day, which corresponds to the defervescence period.

**Table 1:** Demographic information for the study population and the distribution of classifications.

		Traditional				Revised		
	N	DF^#^	DHF^×^	DSS^†^	UC^+^	WS* negative	WS positive	Severe
**Confirmed cases**	267	26	119	46	76	18	142	107
**(%)**	(100)	(9.7)	(44.6)	(17.2)	(28.5)	(6.7)	(53.2)	(40.1)
**Age^#^ (years)**								
**Max**	18	15	14	14	18	16	18	15
**Median**	8	9.5	8	8	8	9	9	8
**Min**	0	0	0	0	0	0	0	0
**Sex^#^**								
**Female**	140	16	65	26	33	8	75	57
**Male**	127	10	54	20	43	10	67	50
**Hospitalization days^#^**								
**Max**	21	6	20	21	17	6	12	21
**Median**	4	4	4	4	3	3	4	5
**Min**	1	1	1	1	1	2	1	1
**Days from fever onset**								
**Max**	13	9	11	10	13	11	11	13
**Median**	5	4	5	5	5	5	5	4
**Min**	0	1	0	2	0	1	0	0
**Previous intravenous hydration**								
**Yes**	191	16	82	41	52	10	98	83
**No**	76	10	37	5	24	8	44	24
**Deaths^#^**	8	-	1	6	1	-	-	8
**ICU^‡#^**	76	-	24	38	14	-	9	67

#dengue fever, **×**dengue hemorrhagic fever, **†**dengue shock syndrome,*warning signs, +unclassified, ‡intensive care unit.

Of the 267 studied cases, 76 (28.4%) received ICU interventions. Fifty-eight of the 76 cases (76.3%) were also part of the 191 children who required continuous monitoring due to hemodynamic instability despite previous fluid management with crystalloids. The recommendations of the International Guidelines for Management of Severe Sepsis [19] were the criteria used by pediatricians to decide on ICU interventions.

The revised scheme classified 97.5% of the patients requiring ICU interventions as severe (67/76), whereas the traditional scheme classified 50% (38/76) of these cases as DSS. All fatal cases received ICU interventions (Table 1).

Signs and symptoms that were frequently observed in the children included in this study are shown in Table 2. No central nervous system or renal dysfunction was observed among the children studied. According to the revised classification scheme, the clinical presentations that defined severe cases were shock (56%, 60/107), respiratory distress (47.6%, 51/107), heart and hepatic dysfunction (12.1%, 13/107), and severe bleeding (21.5%, 23/107) (Table 2). Of the 107 severe cases, 45.8% (49/107) had more than 1 severe manifestation. Among patients with single severe manifestations, 17.7% (19/107) had isolated shock, 12.1% (13/107) had isolated respiratory distress, and 0.93% (1/107) had isolated severe bleeding (data not shown). All cases of organ dysfunction and 87.5% (7/8) of deaths were associated with shock (Table 1 and 2).

**Table 2:** Frequency of laboratory and clinical signs and symptoms according to the World Health Organization 1997 and the 2007 dengue classification schemes.

Clinical signs		Traditional				Revised			
		DF^#^	DHF^×^	DSS^†^	UC^¬^	WS* neg.	WS pos	Severe	
	N	26	119	46	76	18	142	107	
	(%)	(9.7)	(44.6)	(17.2)	(28.5)	(6.7)	(53.2)	(40.1)	
**Decrease in platelet count** ‡	238	12	114	45	67	8	127	103	
	(89.1)	(46.1)	(95.8)	(97.8)	(88.1)	(44.4)	(89.4)	(96.3)	
**Abdominal pain**	189	13	93	37	46	7	92	90	
	(70.7)	(50)	(78.1)	(80.4)	(60.5)	(38.8)	(64.8)	(84.1)	
**Clinical fluid accumulation**	176	-	98	44	34	-	83	93	
	(66)	-	(82.3)	(95.6)	(44.7)	-	(58.4)	(87)	
**Hematocrit change ≥20%**	143	-	68	31	44	-	79	64	
	(53.5)	-	(57.1)	(67.4)	(57.9)	-	(55.6)	(60)	
**Nasal/gum bleeding**	137	12	77	36	12	1	60	76	
	(51.3)	(46.1)	(64.7)	(78.3)	(15.8)	(5.5)	(42.2)	(73.5)	
**Skin paleness**	107	9	41	30	27	3	44	60	
	(40)	(34.6)	(34.4)	(65.2)	(37)	(16.6)	(30.9)	(71)	
**Dehydration**	98	4	45	23	26	5	42	51	
	(36.7)	(15.4)	(37.8)	(50)	(35.5)	(27.7)	(29.6)	(47.6)	
**Liver enlargement**	79	3	40	17	19	-	40	39	
	(29.6)	(5.5)	(33.6)	(37)	(25)	-	(28.2)	(36.4)	
**Erythema**	75	3	35	19	18	4	34	37	
	(28)	(11.5)	(29.4)	(41.3)	(23.7)	(22.2)	(23.9)	(34.6)	
**Shock**	60	-	-	46	14	-	-	60	
	(22.5)	-	-	(100)	(18.4)	-	-	(56)	
**Respiratory distress**	52	-	17	23	12	-	1	51	
	(19.5)	-	(14.3)	(50)	(15.8)	-	(0.7)	(47.6)	
**Lethargy**	45	1	10	28	6	-	6	39	
	(16.7)	(3.8)	(8.4)	(60.8)	(7.9)	-	(4.2)	(36.4)	
**Persistent vomiting**	40	2	15	8	15	-	26	14	
	(15.0)	(7.7)	(13)	(17.4)	(19)	-	(18.3)	(13)	
**Gastrointestinal and/or vaginal hemorrhage**	36	-	25	11	-	-	16	20	
	(13.5)	-	(21)	(24)	-	-	(11.3)	(18.7)	
**Restlessness**	29	-	10	11	8	-	12	17	
	(10.9)	-	(8.4)	(24)	(10.5)	-	(8.5)	(15.8)	
**Severe bleeding ^+^**	23	-	10	13	-	-	-	23	
	(8.6)	-	(6.7)	(28.3)	-	-	-	(21.5)	
**Heart and/or hepatic dysfunction**	13	-	-	13	-	-	-	13	
	(4.9)	-	-	(28.3)	-	-	-	(12.1)	
**Disseminated intravascular coagulation**	8	-	-	8	-	-	-	8	
	(3)	-	-	(17.4)	-	-	-	(7.5)	
**Multiple-organ dysfunction syndrome**	6	-	-	6	-	-	-	6	
	(2.2)	-	-	(13)	-	-	-	(5.6)	

#dengue fever, **×**dengue hemorrhagic fever, **†**dengue shock syndrome, ¬unclassified,*warning signs.

‡ platelet count <50,000/mm^3^, +requiring transfusion of blood products.

Seventy-six (28.5%) patients could not be classified according to the traditional scheme. They did not fulfill all criteria to be classified as DHF or DSS because they did not present with evidence of plasma leakage associated with bleeding manifestation or platelet reduction (Table 2). However, 18.4% (14/76) of these patients had shock and 44.7% (34/76) had clinical evidence of fluid accumulation.

Although these 76 cases could not be classified into any of the categories of the traditional scheme, they were classified by the revised scheme. These patients had the highest frequency of warning signs, including decrease in platelet count (88.1%), abdominal pain (60.5%), a 20% increase in hematocrit concentration (57.9%), clinical fluid accumulation (44.7%), liver enlargement (25%), and persistent vomiting (19%). In total, 65.8% (50/76) of the cases that could not be classified with the traditional scheme were categorized as dengue with warning signs, 25% (19/76) as severe dengue, and 9.2% (7/76) as dengue without warning signs by the revised scheme (data not shown).

The cases that could not be categorized by the traditional scheme were excluded from the sensitivity and specificity analysis to assess their ability to classify severe cases and DSS (n = 183). The revised scheme demonstrated a sensitivity of 86.8% and specificity of 73.0%, while the traditional scheme had sensitivity and specificity of 62.3% and 93.4%, respectively. The sensitivities and specificities of the schemes demonstrated statistically significant differences (*P*<0.05). This significant difference was also observed for positive and negative predictive values (Table 3).

**Table 3:** Difference in sensitivity, specificity, and positive and negative predictive values (PPV and NPV) between the World Health Organization 1997 (traditional) and 2009 (revised) dengue classification schemes.

Sensitivity		*p value (CÏ)*	Specificity		*p value (CI)*
Traditional	Revised		Traditional	Revised	
62.3%	86.8%	<0.001	93.4%	73.0%	<0.001
		(0.12–0.31)			(0.13–0.28)
**PPV** *			**NPV^+^**		
Traditional	Revised		Traditional	Revised	
82.6%	61.6%	<0.001	83.2%	91.7%	0.008
		(1.16–1.55)			(0.85–0.95)

*Positive Predictive Value ^+^Negative Predictive Value; ¨Confidence Interval.

## Discussion

This study demonstrated the superiority of the revised classification (WHO 2009) for the detection of severe cases among hospitalized children with laboratory-confirmed dengue. Similar to the findings of other studies [20], [21], the revised scheme had a significantly better sensitivity (86.8%; *P*<0.001) than the traditional scheme (62.3%) (Table 3). This improved sensitivity allows for better case capture and increased ICU admission, which may aid pediatricians in avoiding deaths due to severe dengue among children. However, it may also result in the misclassification of patients' condition as severe, according to the observed lower positive predictive value (61.6%) when compared with the traditional scheme (82.6%). The lower sensitivity of the traditional scheme (62.3%) is reflected by the presence of 76 unclassified cases (28.5%) that did not present all criteria required to classify them as DHF or DSS, possibly because of the rigidity of the definitions in accounting for early intravenous fluid therapy or the failure to maintain a good record of bleeding.

Pediatricians do not uniformly accept the tourniquet test because it is time-consuming for an epidemic scenario. Whether it would provide additional important information for improving the management of severe children, and if it is specific to either dengue or severity is not well established [22]. Furthermore, the test may be negative during shock syndrome; hence, it would not distinguish DSS from cases of shock without hemorrhage [7].

Although the revised scheme, proposed in 2009, has been described as more effective than the traditional scheme, some controversies remain regarding its specificity, which was lower (73.0%) compared to the specificity of the traditional scheme (93.4%). This difference is perhaps due to a more restricted definition of severity in cases classified as DSS (Table 2). However, some aspects of the new classification also contribute to this lower specificity as the absence of dengue-specific definitions of organ dysfunction, especially for children. Excessive fluid treatment may exacerbate the accumulation of fluid in chest cavities leading to respiratory distress, which qualifies these cases as severe dengue and may explain the proportion of respiratory distress in the absence of shock. The definition of hepatic dysfunction, based only on high levels of transaminases (without an increase in bilirubin or alteration of coagulation), could also overestimate severity. The only case of severe hepatic involvement observed in this study probably occurred because of ischemia, as it was associated with shock [23].

Severe bleeding and respiratory distress symptoms resulted from disseminated intravascular coagulation and acute respiratory distress syndrome, respectively, which were also consequences of shock. Therefore, the hallmark of severe dengue was plasma leakage. Shock followed by organ dysfunction was associated with almost all deaths (7/8) among the studied children. Theoretically, shock could be prevented if plasma leakage and hypovolemia are detected early and managed properly. Nonetheless, the fatality rate was still high (3%) in this group of children even if they had early interventions with fluid management before hospital admission (191/267; Table 1). This study did not assess whether the excess of fluids or inadequate monitoring of clinical progression prior to hospitalization explains the poorer prognosis of some children. The duration of hospitalization was similar across groups and slightly longer for cases of severe dengue (Table 1), probably as a result of complications such as prolonged shock or hypoperfusion for >72 hours (Table 2).

The high observed incidence of warning signs (53.2%) could be related to the identification and utilization of these signs by the pediatricians from Rio de Janeiro as criteria for hospitalization (Table 2) [24]. Warning signs such as the reduction of platelet counts among children, followed by abdominal pain and hemo-concentration in all classification groups, indicates that these signs were the most recognized criteria for hospitalization (Table 2). However, which specific warning signs, if any, may be useful for predicting severity and death in these cases is beyond the scope of this study and would be better assessed with a prospective cohort design.

The major limitation of this study is its retrospective design, because signs and symptoms could have been incompletely recorded, especially among less severe cases, thereby generating a possible classification bias. Nevertheless, the standardized clinical care and rigorous data management protocols used in this study are likely to have mitigated inaccuracy in data collection. Although this study was performed at 3 pediatric reference centers for dengue in the city of Rio de Janeiro, it was a descriptive study, and the lack of a representative sample limits the generalizability of the results.

In spite of these limitations, the study showed the utility of the WHO 2009 dengue classification scheme to detect cases that are not classified by the WHO 1997 dengue classification scheme. In addition, the authors identified issues regarding the specificity of the revised scheme that could be refined in light of standard definitions such as those of the Consensus of Definitions for Organ Dysfunction in Pediatrics [13].

In conclusion, this study demonstrates the better sensitivity of the revised scheme to assess severe cases, which may allow for closer monitoring and management of children, and potentially avoid deaths. Although not easy to apply, it also shows the superiority of the traditional scheme to distinguish the truly severe cases that, in turn, could avoid workload to the health team.

Finally, the pattern of severity among children also permitted us to conclude that the inclusion of unusual dengue manifestations in the revised scheme has not shifted the emphasis from shock with consequent organ dysfunction, the major factor contributing to fatality in this study.
